# Bayesian Compress Sensing Based Countermeasure Scheme Against the Interrupted Sampling Repeater Jamming

**DOI:** 10.3390/s19153279

**Published:** 2019-07-25

**Authors:** Sha Huan, Gane Dai, Gaoyong Luo, Shan Ai

**Affiliations:** 1 School of Physics and Electronic Engineering, Guangzhou University, Guangzhou 510006, China; 2 Advanced Institute of Engineering Science for Intelligent Manufacturing, Guangzhou University, Guangzhou 510006, China

**Keywords:** Bayesian compress sensing (BCS), anti-jammer, target detection

## Abstract

The interrupted sampling repeater jamming (ISRJ) is considered an efficient deception method of jamming for coherent radar detection. However, current countermeasure methods against ISRJ interference may fail in detecting weak echoes, particularly when the transmitting power of the jammer is relatively high. In this paper, we propose a novel countermeasure scheme against ISRJ based on Bayesian compress sensing (BCS), where stable target signal can be reconstructed over a relatively large range of signal-to-noise ratio (SNR) for both single target and multi-target scenarios. By deriving the ISRJ jamming strategy, only the unjammed discontinuous time segments are extracted to build a sparse target model for the reconstruction algorithm. An efficient alternate iteration is applied to optimize and solve the maximum a posteriori estimate (MAP) of the sparse targets model. Simulation results demonstrate the robustness of the proposed scheme with low SNR or large jammer ratio. Moreover, when compared with traditional FFT or greedy sparsity adaptive matching pursuit algorithm (SAMP), the proposed algorithm significantly improves on the aspects of both the grating lobe level and target detection/false detection probability.

## 1. Introduction

The anti-jamming capability of the radar system has played a more and more important role in radar detection under complicated electronic interference circumstances. With the development of digital radio frequency memory (DRFM), interrupted sampling repeater jamming (ISRJ) is extracted with store-and-forward route [1,2]. This mechanism can form several deception targets spreading along the range direction during the tracking process of radar, which leads to false target traction or even a failure in tracking. As a coherent repeater jamming method, ISRJ obtains partial processing gain from pulse compression or coherent integration, thus it can work under a relatively low transmitting power. Typically, ISRJ jammer works within the current radar pulse period, so that the countermeasure approaches based on the inter-pulse diversity become invalid when subjected to ISRJ [3,4,5]. Moreover, various jamming effects could be achieved by a flexible adjustment on the jamming parameters, which will change the power and the distribution of the false targets. Therefore, ISRJ has significant superiorities over other interference manners. More research by using the mentioned method and system of ISRJ has been carried out [6,7,8].

Meanwhile, many electronic counter-countermeasure (ECCM) technologies have been developed to minimize the ISRJ effect [9]. An adaptive transmitting scheme was proposed [10], where waveform optimization was performed to adapt to the jammer perception knowledge. The optimized waveform contains protection pulse orthogonal to the jammer signal at each perceived jammer position. The jammer fragment duration is estimated by the jamming signal bandwidth measurement [11]. Yuan et al. [12] extracted signal segments without jamming based on an energy detection method and exploited these segments to construct the band-pass filter to suppress ISRJ. Inspired by the discontinuous characteristics of the jammer signal in time–frequency (TF) 2D domain of the ISRJ signal, a particular band-pass filter has been designed for jammer suppression [13]. Chen et al. [14] have extended their research on both the anti-jamming performance and the signal-to-ratio (SNR) scope. The ECCM approach based on time-frequency filtering has also been used to the wideband radar [15]. However, the first step of all the ECCM approaches above is to obtain an estimation of the jammer parameter. Low SNR and multi-targets scenario will decrease the estimation accuracy and, at the same time, increase the difficulties on filter construction. Their algorithm performances are also greatly affected by the jamming-to-signal ratio (JSR). 

Wu et al. [16] used a non-periodic interrupted sampling-linear frequency modulated signal (NIS-LFM) which gains benefits of random timing, but there was no in-depth analysis of the anti-jammer performance. A phase-aided distributed compressive sensing (DCS) was developed to suppress the jamming signals with random amplitude and phase [17]. A greedy CS algorithm orthogonal matching pursuit (OMP) was employed in the aforementioned two papers to solve the reconstruction problem [18]. Wei et al. [19] executed sparsity adaptive matching pursuit algorithm (SAMP) for radar detection. These methods do not suffer performance deterioration under high JSR or get an increased complexity in multi-target scenarios. However, the issue of low SNR induced performance degradation has not been addressed.

Although the methods mentioned above have improved the anti-jamming performance to some extent, an efficient and robust countermeasure approach against ISRJ is still desired under high JSR and low SNR circumstance, which is also feasible for both single target and multi-target scenarios. In this paper, we propose a novel countermeasure scheme against ISRJ based on Bayesian compress sensing (BCS) [20,21]. In a typical pulse period, the ISRJ only exists on several designated time segments, and the other time segments partitioned by the repeated jammer in the pulse contain the target signals at the same time they are interference free. The detection capability of the traditional signal processing methods for this kind of incomplete interference-free signal may decrease seriously. However, along with the sparsity of the target distribution, the target signal can be exactly reconstructed with high probability based on the theory of compress sensing (CS) [22]. Hence, a sparse target model is established based on discrete segments without jamming. This model is independent with the jamming power, so it could eliminate the JSR influence. Then, we present a sparse reconstruction algorithm based on BCS. BCS takes additive noise encountered during the compressed measurement into account, which provides a better anti-noise performance. Moreover, a reconstruction optimization solver implemented in alternate iterative manner is presented, whose efficiency is improved by using Fast Fourier transform.

This paper is organized as follows. In Section 2, the ISRJ jamming rejected signal model is built. In Section 3, based on the sparse model of ISRJ jamming rejected signal, the BCS reconstruction algorithm and the corresponding solver are discussed. In Section 5, simulation results are presented to validate the proposed method. 

## 2. ISRJ Jamming Rejected Signal

ISRJ interference usually occurs when surveillance radars track the invading aircraft. The traditional linear frequency modulated signal (LFM) pulse sent by the radar is interfered by the slice forwarding. Here, the ISRJ jamming signal mechanism is briefly discussed, then the model of the ISRJ jamming rejected signal is derived.

### 2.1. Mechanism of ISRJ

The transmitted LFM pulse waveform can be depicted by the following equation

(1) $$S\left(t\right)=\mathrm{rect}\left(\frac{t}{T_{p}}\right)\cdot exp\left[j2\pi (f_{c}t+0.5kt^{2}\right)],$$

where  S(t) is the complex time domain chirp signal in radio frequency (RF) band, $f_{c}$ is the carrier frequency,$\;T_{p}$ is the pulse width and the chirp rate can be defined as $k=B/T_{p}$. B is the entire bandwidth. The function rect(⋅) describes a rectangular function defined as

(2) $$\mathrm{rect}\left(\frac{t}{T}\right)=\{\begin{array}{c} 1\;\;\;\;\;\;\;for\;0\leq t<T\\ 0\;\;\;\;\;\;\;\;\;\;\;\;\;\mathrm{otherwise} \end{array}.$$

**Figure 1:**
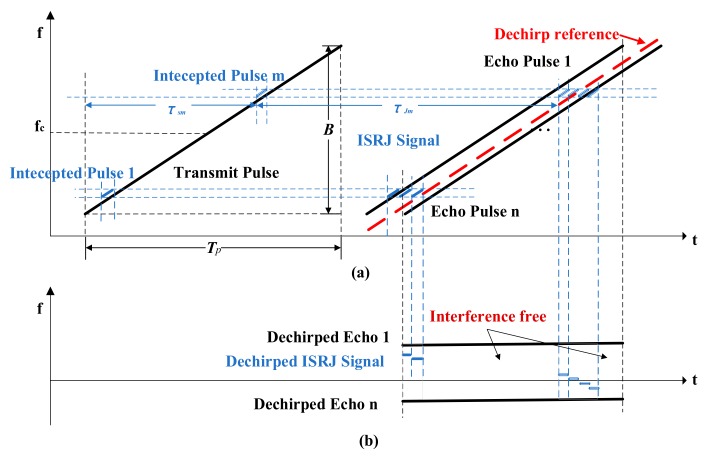
TF characteristics of the transmitted signal and received signals including echo and jamming. (**a**) TF characteristics of the transmitted signal and received signals before de-chirp; (**b**) TF characteristics of the received signals after de-chirp.

The mechanism of ISRJ jamming and the TF characteristics of the echo and jamming signals are shown in Figure 1a. The detected time is aligned with the transmission time for convenience, i.e., the jammer intercepts slices of the detected radar transmitting signal and retransmits them based on set strategy [1,2]. The sampling procedure on the detected radar signal is implemented by $N_{S}$ times in the jammer. The m-th sample time is set at $\tau_{Sm}$ position for $T_{J}$ duration. Then the sampled signals will be stored and retransmitted after $\tau_{Jm}$. The retransmitted signal will keep repeating for $N_{Jm}$ cycles with $\gamma_{mn}$ amplitude constant.

The down conversion along with de-chirp process is accomplished by mixing the received jammed signal with the reference signal. As illustrated in Figure 1b, the de-chirp procedure transforms the echo chirp signals into fixed frequency signals, whose frequency is relevant to the delay corresponding to the targets range and the chirp rate. Therefore, the target range detection can be very facilely converted to a frequency estimation problem, by time–domain windowing and fast Fourier transform (FFT). However, the ISRJ jamming slices will be transformed into several stepped frequency signals, with a period of $N_{Jm}\cdot T_{J}$ at the position of $\tau_{Sm}+\tau_{Jm}$. The step frequency is $-kT_{J}$. In the frequency domain, the echo signals could not be distinguished from the jamming segments, so that it is infeasible to make the interference suppression by the filter. The jamming intervals may destroy the phase information of the echo signals, resulting in a failure in extracting target range information. 

Assuming a constant amplitude reflection $\alpha_{i}$ on each echo, the discrete expression of the de-chirped signal can be written as

(3) $$\begin{array}{c} S_{RE}\left(n\right)=\overset{N_{t}-1}{\underset{i=0}{\sum }}\alpha_{i}\mathrm{rect}\frac{\left(n-\tau_{i}f_{s}\right)}{T_{p}f_{s}}\cdot exp\left[-j2\pi f_{c}\tau_{i}\right]\cdot exp\left[-j\pi k\left(\frac{2n\tau_{i}}{f_{s}}-\tau_{i}^{2}\right)\right]\\ \;+\overset{N_{S}-1}{\underset{m=0}{\sum }}\overset{N_{Jm}-1}{\underset{i=0}{\sum }}\gamma_{mi}\mathrm{rect}\frac{\left[n-f_{s}\left(\tau_{Sm}+\tau_{Jm}+iT_{J}\right)\right]}{T_{J}f_{s}}\cdot \exp\left[-j2\pi f_{c}\left(\tau_{Jm}+iT_{J}\right)\right]\\ \;\cdot \exp\left\{-j\pi k\left[\frac{2n}{f_{s}}\left(\tau_{Jm}+iT_{J}\right)-{\left(\tau_{Jm}+iT_{J}\right)}^{2}\right]\right\}+n_{G}\left(n\right) \end{array}$$

where $f_{s}$ is the sample frequency, $N_{t}$ is the number of reflections, $\tau_{i}=2{ℛ}_{i}/c_{0}$ is the time delay of the i-th reflection with respect to the range ${ℛ}_{i}$, $n_{G}\left(n\right)$ is the discrete additive Gaussian white noise. The specific derivation of (3) can be seen in Appendix A.

### 2.2. Jamming Rejected Signal Model

It is noticed in Figure 1b that the received de-chirped signal is divided into several segments with and without interference. The interference-free segments which only contain the echoes and the noise could be used for targets information extraction. Generally, the transmit power of the jammer is higher than the echo signals, and thus the interference segments can be identified in the time domain through an appropriate power detection method with a suitable energy threshold. The jamming rejected signal model can be obtained when the sample value at the located interference segments is set to be zero, which can be expressed as

(4) $$S_{RE\_c}\left(n\right)=\overset{N_{t}-1}{\underset{i=0}{\sum }}\;{ℛ}_{i}\left(n\right)\cdot \alpha_{\cdot }\cdot exp\left[-\frac{j2\pi k\tau_{\cdot }}{f_{s}}n+\varphi_{i}\right]+ℛ\left(n\right)\cdot n_{G}\left(n\right)\;\mathrm{where}\;{ℛ}_{i}\left(n\right)=\left\{\mathrm{rect}\frac{\left(n-\tau_{i}f_{s}\right)}{T_{p}f_{s}}-\overset{N_{S}-1}{\underset{m=0}{\sum }}\overset{N_{Jm}-1}{\underset{i=0}{\sum }}\mathrm{rect}\frac{\left[n-f_{s}\left(\tau_{Sm}+\tau_{Jm}+iT_{J}\right)\right]}{T_{J}f_{s}}\right\}\;\varphi_{i}=j\pi \left(k\tau_{i}^{2}-2f_{c}\tau_{i}\right).$$

Excluding the second summation term in (3) presenting the jammer signal, the jamming rejected signal is not subject to the jamming power, at the same time, it maintains the original unambiguously measurable range as well as the range resolution because they applied the same time period and sample frequency. This model is not restricted to the point target assumptions, as it includes reflections from multiple targets as well as multiple reflections from a single target.

However, it should be noted that the jamming rejected signal with an incomplete structure is not optimal for conventional FFT processing due to the information defect on jamming sections. It will lead to relatively high, range independent spurs/residues in the range profile, masking weaker targets. The residues are unlike normal sidelobes that decrease with increasing distance to the peak, it remains nearly constant at a certain level of below the target peak based on the jamming-free duty ratio and the position of the jamming segments.

To overcome this obstacle, novel algorithms must be applied to reconstruct the target detection using the incomplete distributed signal. Since the jamming rejected signal could be regarded as a non-equidistant subsampling in the time domain, and the radar scene is typically sparse, the area observed consists only of a few dominant targets besides clutter. It is a basic prerequisite for CS reconstruction. The sparse signal model and reconstructed algorithm will be discussed in detail in the following section.

## 3. Sparse Reconstruction Algorithm Based on BCS

In this section, we build the sparse model of the jamming rejected signal based on (4) at first. Then, cost function based on BCS is established. In the end, an efficiency solver is illustrated to implement the proposed algorithm. 

### 3.1. Sparse Model for Jamming Rejected Signal

The radar targets can be regarded as the contribution of several limited strong scattering centers, which are sparse along the range domain. As mentioned above, the target range is proportional to the de-chirped echo frequency, it can be expressed as a sparse weight vector $\theta \in {\mathbb{C}}^{N}$, in the frequency domain. Equation (4) can be rewritten as a matrix form

(5) s=P⋅F⋅θ+n,

where $n\in {\mathbb{C}}^{M}$ denotes the additive complex Gaussian noise vector with independent and identical distribution. $s\in {\mathbb{C}}^{M}$ stands for the jamming rejected sample vector. F is the standard Fourier matrix in size N×N, it is given by

(6) $$F=\left[\begin{array}{ccc} \begin{array}{cc} 1 & 1\\ 1 & \omega^{1} \end{array} & \cdots & \begin{array}{c} 1\\ \omega^{\left(N-1\right)} \end{array}\\ \vdots & \ddots & \vdots \\ \begin{array}{cc} 1 & \omega^{\left(N-2\right)}\\ 1 & \omega^{\left(N-1\right)} \end{array} & \cdots & \begin{array}{c} \omega^{\left(N-2\right)\left(N-1\right)}\\ \omega^{\left(N-1\right)\left(N-1\right)} \end{array} \end{array}\right]\;,$$

where ω=exp{−j2π/N}.

The matrix $P\in {\mathbb{C}}^{M\times N}$ represents the un-jammed position of the received signal. M denotes the number of the unjammed sample points, and P is a partial unit matrix. The lines correspond to the position of the zero elements in ℛ(n) which is eliminated. Define the measurement matrix $F_{p}=P\cdot F$**,** which is a typical partial Fourier matrix. Rows in $F_{p}$ are sampled from F according to P by rows. In the compress sensing theory, if θ is sparse in the frequency domain, and the measure matrix $F_{p}$ satisfies the restricted isometry property (RIP) condition, θ can be reconstructed from a degrading dimensional observation matrix by solving a sparse constrained and ill-posed problem.

Many compress sensing algorithms have been proposed to solve ill-posed problems [23]. Some greedy strategies such as Matching pursuit (MP) iteratively select the basis vector to realize sparse recover [24]. Series of basis pursuit (BP) methods focus on the convex optimization after $\ell_{1}-$norm relaxation [25]. However, the solution of the previous version of CS-based methods may not be sparse enough, or the regularized parameter should be tuned manually, which is undesirable in practical application. Besides, the performance of the approaches degrades dramatically on the noisy and cluttered scenes.

BCS is demonstrated to be equivalent to an iterative reweighted $\ell_{1}$ minimization [26]. In comparison to the BP method based on $\ell_{1}$ penalty term, the Bayesian formalism adopted posterior estimation can be more approached to the sparse optimal solution. In addition, BCS takes account for additive noise encountered when executing the compressed measurement, which guarantees a better anti-noise performance. Hence, we select the BCS algorithm to reconstruct the range signal based on the interference-free time segments. 

### 3.2. Reconstruction Optimization with BCS

The real and imaginary parts of the complex Gaussian noise n in (5) are equal and independent, denoted by $n_{r}$ and $n_{i}$, respectively. They both have zero mean and follow Gaussian distributions with variance $\sigma^{2}$. The probability density function is given as follows

(7) $$P\left(n|\sigma^{2}\right)=\left[{\left(2\pi \sigma^{2}\right)}^{-\frac{M}{2}}exp\left(-\frac{1}{2\sigma^{2}}||n_{r}||{}_{2}\right)\right]\cdot \left[{\left(2\pi \sigma^{2}\right)}^{-\frac{M}{2}}exp\left(-\frac{1}{2\sigma^{2}}||n_{i}||{}_{2}\right)\right]\;=\left[{\left(2\pi \sigma^{2}\right)}^{-M}exp\left(-\frac{1}{2\sigma^{2}}||n||{}_{2}\right)\right].$$

$||\cdot ||{}_{2}$ denotes the Euclidean norm of the vector, which is the square root of the sum of squares. Therefore, the Gaussian likelihood model of the jamming rejected samples can be derived as

(8) $$P\left(s|\theta ,\sigma^{2}\right)=\left[{\left(2\pi \sigma^{2}\right)}^{-M}exp\left(-\frac{1}{2\sigma^{2}}||s-F_{p}\cdot \;\theta {||}_{2}\right)\right].$$

The sparsity of θ can be characterized by setting a sparseness-promoting prior. Generally, Laplace density function is used to represent the sparseness of θ in BSC theory, which is

(9) $$P\left(\theta |\gamma \right)={\left(\frac{\gamma }{2}\right)}^{N}exp\left(-\gamma ||\theta {||}_{1}\right),$$

where γ is the scale coefficient of the Laplace density function, and $||\cdot ||{}_{2}$ is ℓ1−norm  simply the sum of the absolute values of the vector elements. Then, the range detection now turns to an estimation of θ from a noisy sampled sequence s. We now concentrate on seeking the value $\hat{\theta }\left(s\right)$ which maximize the posterior P(θ|s), that is

(10) $$\hat{\theta }\left(s\right)=\arg\;\underset{\theta \in {\mathbb{C}}^{N}}{\max}\left[P\left(\theta |s\right)\right]$$

with the help of the Bayes’ Rule

(11) $$P\left(\theta |s\right)=\frac{P\left(s|\theta \right)\cdot P\left(\theta \right)}{P\left(s\right)}.$$

P(s) above has no direct functional dependence on the parameter θ with respect to which we want the right-hand side of (10) to be maximized. After dropping the denominator of (11), the formula can be written as

(12) $$\hat{\theta }\left(s\right)=\arg\;\underset{\theta \in {\mathbb{C}}^{N}}{\max}\left[P\left(s|\theta ,\sigma^{2}\right)\cdot P\left(\theta |\gamma \right)\right]$$

with the maximum likelihood (ML) estimation, we use the logarithm of the posteriors to make this problem simple as

(13) $$\hat{\theta }\left(s\right)=\arg\;\underset{\theta \in {\mathbb{C}}^{N}}{\max}\left\{\log\left[P\left(s|\theta ,\sigma^{2}\right)\right]+\log\left[P\left(\theta |\gamma \right)\right]\right\}.$$

Substitute (8) and (9) into (13), the maximum a posteriori method (MAP) estimator turns to be

(14) $$\begin{array}{ll} \hat{\theta }\left(s\right) & =arg\;\underset{\theta \in {\mathbb{C}}^{N}}{\max}\left\{-\frac{1}{2\sigma^{2}}||s-F_{p}\cdot \theta {||}_{2}-\gamma ||\theta {||}_{1}\right\}\\ & =arg\;\underset{\theta \in {\mathbb{C}}^{N}}{\min}\left\{||s-F_{p}\cdot \;{||}_{2}+\lambda ||\theta {||}_{1}\right\}, \end{array}$$

where $\lambda =2\sigma^{2}\gamma$ is the feature weight parameter, proportional to the noise variance and the Laplace scale coefficient. By making a reasonable estimation of the weight parameter λ, the performance of the sparsity reconstruction algorithm can be ensured. The ℓ2−norm on the right-hand side of (14) preserves the corresponding relation between s and $\hat{\theta }$**.** The ℓ1−norm constraints that limited non-zero points exist in $\hat{\theta }$.

Generalized cross validation (GCV) can be used to choose an appropriate value of λ in a data-driven way [27,28], which will approximately minimize the expected value of the predictive risk. It could provide an estimate for λ without requiring the noise variance. The GCV estimate of λ is the minimizer of

(15) $$GCV_{\lambda }=\frac{\frac{1}{N}||F_{p}\cdot \hat{\theta_{\lambda }}-s{||}_{2}^{2}}{{\left[\frac{1}{N}tr\left(I-K_{\lambda }\right)\right]}^{2}},$$

where $\hat{\theta_{\lambda }}$ denotes the solution obtained by using λ, tr(⋅) is the matrix trace function, $I\in {\mathbb{R}}^{N\times N}$ is an identity matrix. $K_{\lambda }$ is the influence matrix defined by

(16) $$F_{p}\hat{\theta_{\lambda }}=K_{\lambda }s.$$

The golden section method [29] can be used to search the value of λ which can minimize $GCV_{\lambda }$ within a given interval.

### 3.3. Efficient Solver to the Reconstruction Optimization

Several methods are available to solve the local minima of (14) when parameter λ has been chosen. A solver base on Quasi-Newton is presented here for $\hat{\theta }$ which presents the target range information [30]. The discontinuousness problem of the ℓ1−norm could be solved by a reasonable approximation below

(17) $$||\theta \left(n\right){||}_{1}\approx {\left({\left|\theta \left(n\right)\right|}^{2}+\tau \right)}^{\frac{1}{2}}.$$

The parameter τ should be set relatively small and nonnegative, and thus the expression on the right side of the equation can approach to the ℓ1−norm. Under the approximation, the MAP estimator can be reformatted as

(18) $$\hat{\theta }\left(s\right)=arg\;\underset{\theta \in {\mathbb{C}}^{N}}{\min}\left\{f\left(\theta \right)\right\},$$

where $f\left(\theta \right)=||s-F_{p}\cdot \;\theta {||}_{2}+\lambda \sum_{n=0}^{N-1}{\left({\left|\theta \left(n\right)\right|}^{2}+\tau \right)}^{\frac{1}{2}}$.

The conjugate gradient function of f(θ) can be calculated to be

(19) $$\nabla_{\theta^{*}}f\left(\theta \right)=2F_{p}^{H}F_{p}\theta +\lambda U\left(\theta \right)\theta -2F_{p}^{H}s.$$

U(θ)θ depicts the derivation result of the Summation term in f(θ), which can be written as

(20) $$U\left(\theta \right)=\mathrm{diag}\left[{\left({\left|\theta_{0}\right|}^{2}+\tau \right)}^{-\frac{1}{2}},{\left({\left|\theta_{1}\right|}^{2}+\tau \right)}^{-\frac{1}{2}},\cdots ,{\left({\left|\theta_{N-1}\right|}^{2}+\tau \right)}^{-\frac{1}{2}}\right].$$

Define the N×N matrix H(θ), consisting of information of the Hessian.

(21) $$H\left(\theta \right)=2F_{p}^{H}F_{p}+\lambda U\left(\theta \right)$$

It is obvious that H(θ) depends on the objective variable θ. The minimal searching in (18) now turns to solve the equation below:

(22) $$\theta =2H^{-1}\left(\theta \right)F_{p}^{H}s$$

where H(θ) is defined by the parameter θ which needs to be estimated. An alternate iteration solver is presented to solve the formula above. As that the matrix H(θ) inversion requires complex calculation, the conjugate gradient algorithm (CGA) is applied in the inner layer iteration to solve ${\hat{\theta }}^{\left(g+1\right)}$ in (23). In CGA, the matrix inversion calculation of H(θ) is replaced by performing the calculation on the secant equation

(23) $$H\left({\hat{\theta }}^{\left(g_{n}\right)}\right){\hat{\theta }}^{\left(g_{n+1}\right)}=2F_{p}^{H}s.$$

The specific algorithm is described in Algorithm 1.
**Algorithm 1** Alternate Iteration Optimization for BCS Reconstruction **Input**: The jamming rejected sample vector s, the measurement matrix $F_{p}$, the threshold for the iteration solver ρ; **Output**: Reconstructed sparse solution ${\hat{\theta }}^{\left(g_{n}\right)}$.**Procedure**: **Step 1**: Initialize ${\hat{\theta }}^{\left(g_{0}\right)}=Fs,$ set cycle index n=0; **Step 2**: Update $H\left({\hat{\theta }}^{\left(g_{n}\right)}\right)$ according to Equation (21); **Step 3**: Use CGA to optimize Equation (23) based on $H\left({\hat{\theta }}^{\left(g_{n}\right)}\right)$, and obtain ${\hat{\theta }}^{\left(g_{n+1}\right)}$; **Step 4**: If ${\left|{\hat{\theta }}^{\left(g+1\right)}-{\hat{\theta }}^{g}\right|}_{2}/{\left|{\hat{\theta }}^{g}\right|}_{2}\leq \rho$, stop the iteration and output the sparse solution ${\hat{\theta }}^{\left(g_{n+1}\right)}$; otherwise let n=n+1 and back to Step 2.

The computational complexity could be reduced taking into consideration that the measurement matrix is a partial Fourier matrix. FFT is applied to implement the matrix multiplication of $F_{p}^{H}F_{p}{\hat{\theta }}^{\left(g+1\right)}$ which occupies the major computational load in the multiple iterative calculations of CGA. As $F_{p}^{H}F_{p}{\hat{\theta }}^{\left(g+1\right)}$ is equivalent to $FP_{N}F^{H}{\hat{\theta }}^{\left(g+1\right)}$, $P_{N}$ is a unit matrix with zero elements corresponding to the position of the non-zero elements in ℛ(n). The matrix multiplication can be completed by performing the inverse FFT on ${\hat{\theta }}^{\left(g+1\right)}$, then setting some lines to zero based on $P_{N}$, and running an *N* point FFT at last. $F_{p}^{H}s$ can be executed in the same way. 

We resort to Monte Carlo experiments in the simulation stage to evaluate the detection performance in the next section. SAMP will be applied as a comparison [31]. It is also a greedy algorithm based on compress sensing theory, but SAMP does not require inputting the number of iterations based on the sparsity of the signal, unlike the OMP algorithm.

## 4. Simulation

In this section, simulations are carried out to demonstrate the proposed algorithm, revealing an improved detection performance. 

We set simulation parameters for a typical LFM surveillance radar system. In the simulation settings, ISRJ jammer intercepts the transmitted signal and retransmits it repeatedly with a delay. All variables in the jamming process are parameterized. The received signal including the echo signals and the ISRJ jamming will be de-chirped and converted to baseband. Assuming the interference can be completely detected and eliminated in the time domain, the remaining discrete interference-free samples are utilized for target reconstruction. 

The simulation parameters are listed in Table 1. A Hamming window is applied to the both BCS and SAMP recovered target signal to make a fair comparison corresponding to the reference signal result. Although it would not affect the reconstruction performances of the proposed algorithm or the contrast algorithm, the main lobe width of the adding window was taken into account in the following analysis.

In order to investigate the reconstruct performance vs. the jamming-free duty ratio, we denote parameter η as the formula below. η presents the sparsity degree of the signal samples, these samples constitute an incomplete date set without jamming.

(24) $$\eta =\frac{T_{p}-\sum_{m=0}^{N_{S}-1}N_{Jm}\cdot T_{J}}{T_{p}}$$

### 4.1. Performance of the Jamming Rejected Signal

Simulation is carried out to compare the effect of the ISRJ jamming and jamming rejection on the range detection performance. The amplitude of the different jamming segments $\gamma_{m\cdot }$ is set as a constant. During the pulse period, the jammer generates six segments of interference, the position $\tau_{Sm}$ and repeat starting time $\tau_{Jm}$ of which are random. 

**Figure 2:**
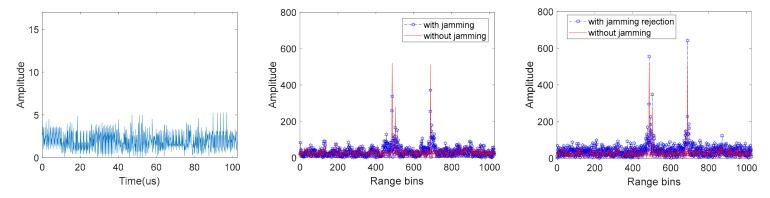

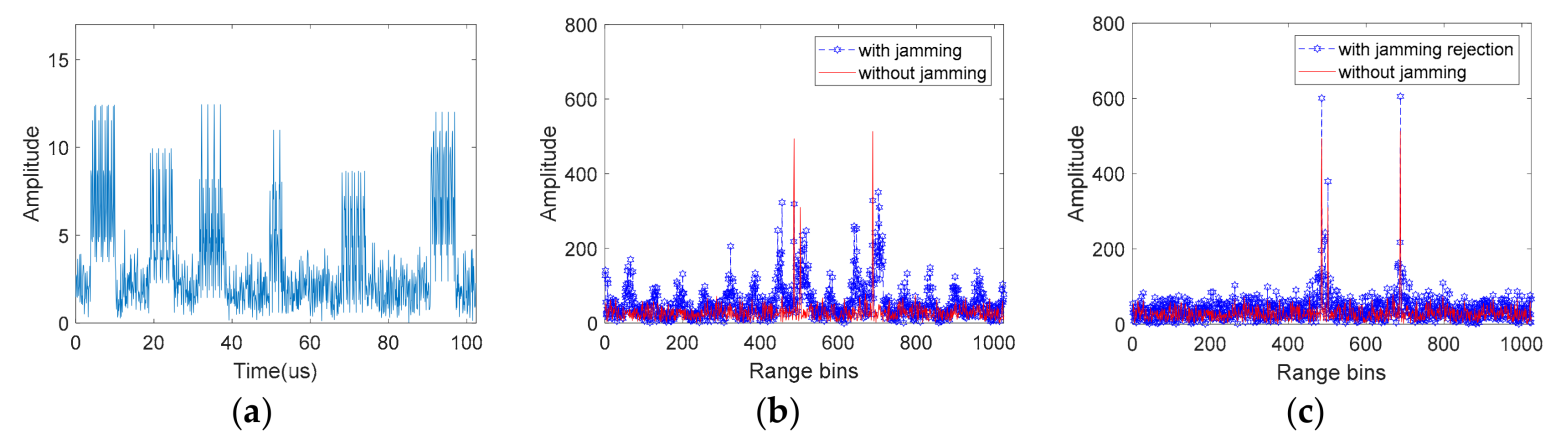
The waveform of the received signal and the range compression results with different jamming-to-signal power ratio. (**a**) Amplitude of the received signal with jamming; (**b**) range compression result of the received signal with jamming; (**c**) range compression result of the received jamming rejected signal. (From top to bottom, the jamming-to-signal power ratio are 0dB and 10dB respectively).

A reference signal with no jamming is applied to compare the performance of various waveforms in Figure 2. Figure 2a gives an intuitive impression of the waveforms with different jamming power. The interference can be identified from the amplitude domain when the jamming power is high. Range performance of the received signals with jamming illustrates a sharp deterioration when the jamming power gets higher in Figure 2b. Meanwhile, the FFT range compression results of the jamming rejected signals in Figure 2c are not deteriorated with the increase of the jamming power.

### 4.2. Reconstruction Performance

To estimate the ability of the reconstruction algorithm on detecting small adjacent signals with the presence of large signals, we consider example scenarios with three scattering targets in different range. The first and second targets are close, and the power of the second target echo signal is 6 dB less than the other two targets. A total of six ISRJ jamming segments are added on the echo signal. $N_{J}$ is set to 6, and the duration *T_J_* are [6.25, 3.125, 6.25, 6.25, 6.25, 6.25] (μs) and [12.5, 12.5, 6.25, 12.5, 12.5, 12.5] (μs), respectively, correspond to η=65.7% and η=31.3%. The inner iteration threshold of the BCS algorithm is set to $10^{-3}$ while the outer threshold is $\rho \;=\;10^{-2}$. The step size of the SAMP method is three and the threshold is set to 0.3. A limitation of the SAMP iteration number is set to less than 200 to prevent redundancy.

**Figure 3:**
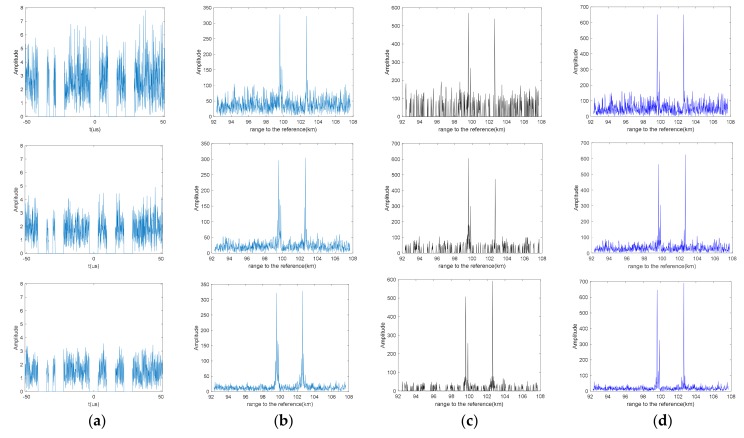
The waveform of the jamming rejected signal (**a**) and the reconstruction results of tradition FFT (**b**), sparsity adaptive matching pursuit algorithm (SAMP) (**c**), Bayesian compress sensing (BCS) (**d**), separately on η=65.7% with different signal-to-noise ratio (SNR). (From top to bottom, the signal-to-noise ratio of the echo signals are SNR=−5 dB, SNR=0 dB, SNR=10 dB, respectively).

Figure 3 demonstrates the reconstruction performance of tradition FFT, SAMP, and BCS on the jamming rejected signals. On the condition of η=65.7%, the tradition FFT result shows relative high grating lobe on both sides of the target main lobe, even higher than the main lobe of the weak target. The mechanism of the greedy algorithm leads to no grating lobe problem in SAMP. However, when the SNR gets lower, some undesirable peaks will appear on the random positions along the frequency axis which can be regarded as a clutter signal. The BCS algorithm on the (d) column successfully reconstructs the three targets under three kinds of SNR condition. At the same time, it realizes a great improvement on the level of the grating lobe compared to the tradition FFT. 

**Figure 4:**
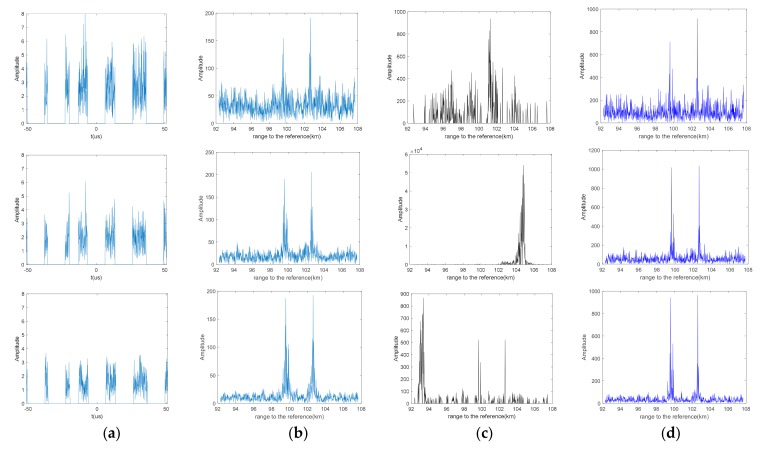
The waveform of the jamming rejected signal (**a**) and the reconstruction results of tradition FFT (**b**), sparsity adaptive matching pursuit algorithm (SAMP) (**c**), Bayesian compress sensing (BCS) (**d**), separately on η=31.3% with different signal-to-noise ratio (SNR). (From top to bottom, the signal-to-noise ratio of the echo signals are SNR=−5 dB, SNR=0 dB, SNR=10 dB, respectively).

The reconstruction performance of the three methods above with increasing jamming signal is shown in Figure 4. In FFT and BCS simulation, the three targets can still be distinguished in the reconstructed frequency domain; however, the levels of the grating lobe both get raised compared to the results in the lower jammer ratio. SAMP could not recover the target range information when η gets low. Even though three peaks will appear at the target position after SAMP when SNR = 5 dB, there are still some undesirable and strong clutters along the range. 

**Figure 5:**
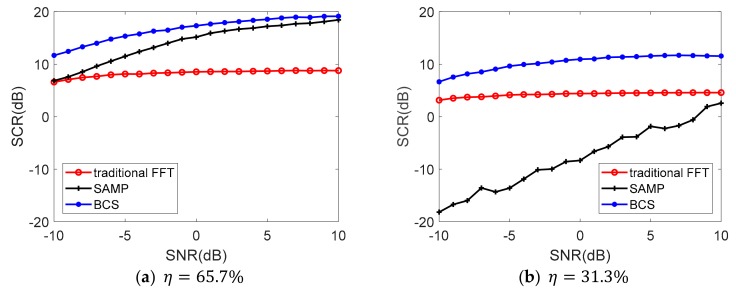
The SCR versus different SNR of the echo signal.

**Figure 6:**
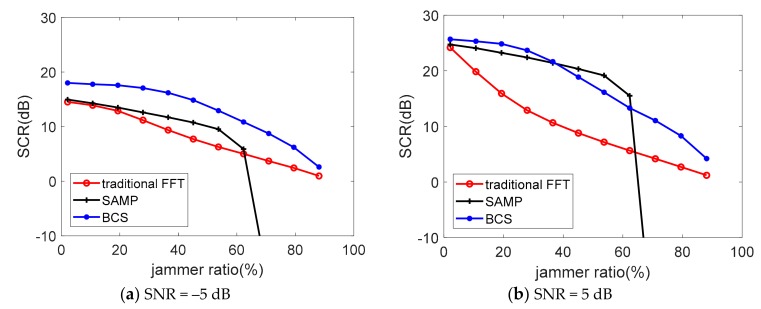
The SCR versus different jammer ratio of the echo signal.

Considering there is no analytic solution for the proposed algorithm or the comparison algorithm, the Monte Carlo method is introduced here to further examine the reconstruction performance with different SNR and jammer ratio. peak side lobe ratio (PSLR) and mean square error (MSE) are usually used as indicators for the quality of radar image reconstruction [30]. Then, in the background of target detection and tracking, in addition to successful reconstruction, we also wondered whether reconstruction will introduce false targets. Some undesired peaks will appear after reconstruction which can be regarded as clutters. Therefore, we used signal-to-clutter ratio (SCR) after reconstruction as one of the evaluation indicators of the reconstruction performance. It not only can reflect the power ratio of the grating lobe in FFT and BCS, but could also weigh the clutter level in SAMP. A total of 1000 Monte Carlo results of the contrast on SCR based on different SNR and jammer ratio is demonstrated in Figure 5 and Figure 6. The numeric comparison of the reconstruction performance is listed in Table 2.

In Figure 5, the SCR performance of SAMP and BCS both show better performance than the traditional method. BCS performs the best among the SNR range under both jammer ratios. We can still conclude that SAMP is more sensitive to the noise for the bigger slope of the SCR data line. The SCR of BCS is comparatively stable when SNR differs. This is because the BCS algorithm contains a noise model when modeling.

In Figure 6, the SCR levels decrease when jammer ratio gets higher for all the three algorithms. BCS performs best when SNR = −5 dB in all the ratio conditions, even when only 10% of the sample data is available. The SAMP outperforms BCS when the jammer ratio between 36.5%–62.3% under SNR = 5 dB in Figure 6b. SAMP perform better only when SNR is relatively high and the valid signal ratio locates in a specific interval, but when the valid signal ratio decreases, based on the maximal correlation principle, many wrong coordinates are admitted into the recover list leading to a sharp decline in SCR [31]. The simulation result shows that the SAMP turns invalid when the useful sample date ratio is less than 30%.

From Table 2 it is clearly observed that the BCS algorithm is stable with little fluctuation in reconstructed results. Therefore, the BCS reconstruction algorithm exhibits strong robustness both on SNR and jammer ratio based on the SCR evaluation index.

### 4.3. Target Detection 

The above three algorithms are followed with a cell-averaging constant false alarm rate (CA-CFAR) detector to characterize the performance of target detection [32]. The detection probability (P_d_) and the probability of false detection (P_fd_) are simulated with the probability of false alarm $P_{FA}=10^{-4}$ [33]. The noise is modified on the echo signal based on SNR, assuming the radar echo intensity is stable. 

**Figure 7:**
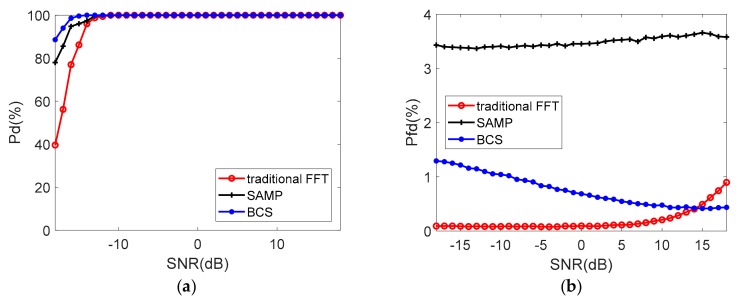
The probability of detection (**a**) and false detection (**b**) (single target, η=65.7%).

In the single target scenario, with lower interference duty cycle, Figure 7 presents that all the three algorithms get 100% target detection probability when SNR is greater than −12 dB. BCS algorithm shows higher success rate compared to the traditional FFT method and SAMP when SNR is lower than −12 dB. At the same time, P_fd_ rate of BCS is slightly higher than the preset $P_{FA}$ because the noise power no longer obeys Rayleigh distribution after sparse reconstruction. The probability of false detection of SAMP is much higher than the BCS.

**Figure 8:**
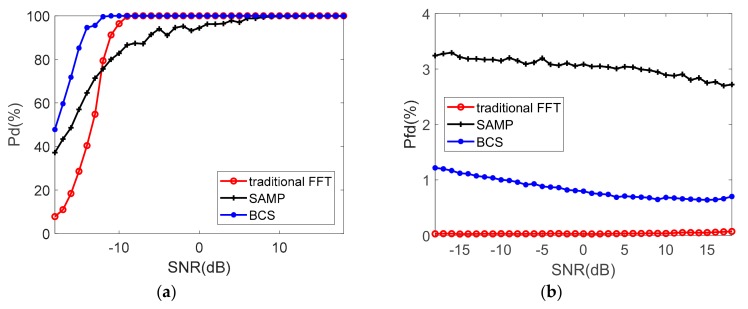
The probability of detection (**a**) and false detection (**b**) (single target, η=31.3%).

As the interference duty cycle rises, the available sample number decreases. In Figure 8, 100% target detection probability can be achieved by FFT and BCS when SNR is greater than −9 dB. BCS is possible to achieve radar detection rates greater than 90% above the received SNR of −14 dB, and it is possible to achieve $P_{d}>99.99\%$ for received SNR above −11 dB. The performance of SAMP declines rapidly when η gets lower. It could not realize a stable detection even when SNR is relatively high. The tradition FFT performs the worst when SNR decreases. Meanwhile, the $P_{d}$ rate of BCS gets a bigger advantage than that of SAMP. The false detection probability of BCS deteriorates slightly to the simulation results in Figure 7 when η decreases.

**Figure 9:**
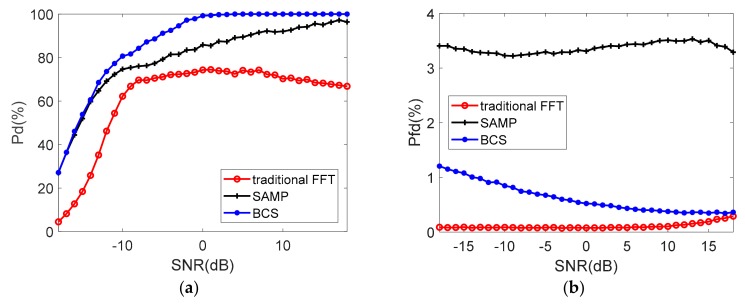
The probability of detection (**a**) and false detection (**b**) (three targets, η=65.7%).

In the three-target and less jammer scenario, because the grating lobe of the strong target conceals the main lobe of the weak target, traditional FFT and SAMP shows invalidation at detecting the weak target by 100% even in relatively high SNR in Figure 9. BCS algorithm keeps a stable detection performance on all the three targets when SNR increases.

## 5. Conclusions

In this paper, a novel countermeasure scheme is proposed against the ISRJ jammer, which uses the discontinuous unjammed time segments to reconstruct the target detection signal based on BCS. An efficient alternate iteration is applied to optimize and solve the MAP of the sparse targets model. Numerical simulation indicates strong robustness of the proposed approach over a relatively large range of SNR and jammer ratio for both single target and multi-target scenarios. In addition, when compared with traditional FFT or greedy algorithm SAMP, BCS reconstruction algorithm demonstrates an improved performance on both the grating lobe level and the target detection/false detection probability. Our further research is to establish a reliable noise distribution model after BCS reconstruction for setting an appropriate CFAR detection threshold. 

## Figures and Tables

**Table 1:** Simulation parameter.

**Radar parameters**	Bandwidth (*B*)/MHz	4
	Pulse width ($T_{p}$)/us	100
	Carrier frequency ($f_{c}$)/GHz	3
	Sample frequency ($f_{s}$)/MHz	10
**Target parameters**	Target number ($N_{t}$)	3
	Target range ($R_{t}$)/Km	[99.5, 99.8, 103.2]

**Table 2:** Reconstructed signal-to-clutter ratio (SCR) performance with different SNR and ratio (dB) ^1^.

SNR (dB)	η	FFT	SAMP	BCS
−5	65.7	8.1282 (0.0018)	11.5084 (0.0025)	15.3455 (0.0024)
0		8.5437 (0.0012)	15.1487 (0.0029)	17.3262 (0.0027)
5		8.6926 (0.0007)	17.2104 (0.0035)	18.5484 (0.0021)
−5	31.3	4.1401 (0.0013)	−13.5929 (0.0311)	9.6366 (0.0032)
0		4.4162 (0.0009)	−8.3397 (0.0330)	10.9439 (0.0024)
5		4.5387 (0.0005)	−1.8454 (0.0228)	11.5521 (0.0015)

^1^ Data are mean (standard deviation).

## Appendix A

The received signal consists of the echo signal reflected from the targets and ISRJ jamming signals. The echo signals can be written as

(A1) $$S_{echo}\left(t\right)=\overset{N_{t}-1}{\underset{i=0}{\sum }}\alpha_{i}S\left(t-\tau_{i}\right)=\overset{N_{t}-1}{\underset{i=0}{\sum }}\alpha_{i}\mathrm{rect}\frac{\left(t-\tau_{i}\right)}{T_{p}}\cdot exp\left[j2\pi f_{c}\left(t-\tau_{i}\right)]\cdot exp[j\pi k{\left(t-\tau_{i}\right)}^{2}\right]$$

The ISRJ jamming signal can be described as

(A2) $$S_{jam}\left(t\right)=\overset{N_{S}-1}{\underset{m=0}{\sum }}\overset{N_{Jm}-1}{\underset{i=0}{\sum }}\gamma_{mi}\mathrm{rect}\frac{\left(t-\tau_{Sm}-\tau_{Jm}-iT_{J}\right)}{T_{J}}\cdot exp\left[j2\pi f_{c}\left(t-\tau_{Jm}-\cdot T_{J}\right)]\;\cdot exp[j\pi k{\left(t-\tau_{Jm}-iT_{J}\right)}^{2}\right]$$

Therefore, the received signal is given as the following expression

(A3) $$S_{RE}\left(t\right)=S_{echo}\left(t\right)+S_{jam}\left(t\right)+n\left(t\right),$$

where *n*(*t*) is the continuous additive Gaussian white noise. The received baseband signal can be expressed as (6), which is illustrated in Figure 1b.

(A4) $$\begin{array}{c} S_{RE_{b}}\left(t\right)=S_{RE}\left(t\right)\cdot exp\left[-j2\pi (f_{c}t+0.5kt^{2}\right)]\\ =\overset{N_{t}-1}{\underset{i=0}{\sum }}\alpha_{i}\mathrm{rect}\frac{\left(t-\tau_{i}\right)}{T_{p}}\cdot exp\left[-j2\pi f_{c}\tau_{i}\right]\cdot exp\left[-j\pi k\left(2t\tau_{i}-\tau_{i}^{2}\right)\right]\\ \;+\overset{N_{S}-1}{\underset{m=0}{\sum }}\overset{N_{Jm}-1}{\underset{i=0}{\sum }}\gamma_{mi}\mathrm{rect}\frac{\left(t-\tau_{Sm}-\tau_{Jm}-iT_{J}\right)}{T_{J}}\cdot \exp\left[-j2\pi f_{c}\left(\tau_{Jm}+iT_{J}\right)\right]\\ \;\cdot \exp\left\{-j\pi k\left[2t\left(\tau_{Jm}+iT_{J}\right)-{\left(\tau_{Jm}+iT_{J}\right)}^{2}\right]\right\}+n_{G}\left(t\right) \end{array}$$

After replacing t in (A4) with $\frac{n}{f_{s}}$, we get the discrete expression of $S_{RE_{b}}\left(t\right)$ in (3).
